# Exploring the biological functions of PCOS: identifying hub androgen-related genes through bioinformatics

**DOI:** 10.3389/fmed.2026.1693216

**Published:** 2026-03-19

**Authors:** Xinling He, Lan Su, Ji Yin, Yuanyuan Lai, Han Yang, Zheng Yu, Xiaoyan Zheng, Jia Liu, Jie Yang

**Affiliations:** 1 Acupuncture and Tuina School, Chengdu University of Traditional Chinese Medicine, Chengdu, China; 2 Division of Internal Medicine, West China Hospital, Institute of Integrated Traditional Chinese and Western Medicine, Sichuan University, Chengdu, China; 3 School of Intelligent Medicine, Chengdu University of Traditional Chinese Medicine, Chengdu, China; 4 West China Second University Hospital of Sichuan University, Chengdu, China; 5 Department of Traditional Chinese medicine, West China Second University Hospital of Sichuan University, Chengdu, China

**Keywords:** Polycystic ovary syndrome, androgen, hub gene, consensus cluster, predictive model

## Abstract

Polycystic ovary syndrome (PCOS) is a prevalent reproductive endocrine disorder in women. While the role of androgens in PCOS is well-recognized, the underlying mechanisms warrant further investigation. In this study, we identified potential hub androgen-related genes (ARGs) in PCOS and established diagnostic and classification models to find novel biomarkers for PCOS therapy. Five datasets (GSE34526, GSE80432, GSE95728, GSE124226, and GSE137684) were retrieved from GEO, followed by data normalization and batch effect removal. To identify hub genes, a PPI network was constructed based on differentially expressed ARGs. Subsequently, we employed the least absolute shrinkage and selection operator (LASSO) regression analysis and random forest (RF) algorithm to screen hub ARGs. Besides, hub ARGs associated with PCOS were determined by integrating the results of the three algorithms. Additionally, a nomogram was constructed using these hub ARGs to predict the risk of PCOS development. We also investigated the classification of ARG molecular subtypes and assessed immune characteristics and gene expression profiles in different subtypes. Lastly, RT-qPCR was utilized to validate the reliability of the hub genes. A total of 91 ARGs were retrieved from the GSEA website. This study included 26 healthy and 34 PCOS samples. Using the LASSO identified 13 key ARGs, RF identified 10 crucial ARGs, and PPI identified 19 pivotal ARGs. Integration of three methods identified four hub ARGs (*ALDH1A1, DHRS9, PRKCB*, and *SGPL1*). And a nomogram was constructed to predict the risk of PCOS occurrence. Notably, we validated the expression levels of the 4 hub ARGs in ovarian tissues from PCOS mice using RT-qPCR. The results showed that the expression levels of *DHRS9, SGPL1*, and *ALDH1A1* were significantly downregulated, while *PRKCB* was significantly upregulated, which was consistent with our data analysis findings. Furthermore, samples were divided into two distinct ARG patterns and further explored the relationship between immune cell infiltration and these patterns. ARG scores were significantly higher in cluster A or gene cluster A compared to cluster B or gene cluster B. Finally, we evaluated the expression levels of PCOS-related genes in distinct clusters. In summary, our results may further elucidate the mechanisms of PCOS pathogenesis and offer novel ideas for PCOS diagnosis and treatment.

## 1 Introduction

Polycystic ovary syndrome (PCOS) is one of the most common endocrine disorders in women of reproductive age, characterized by ovulatory dysfunction, hyperandrogenemia (HA), and polycystic ovary morphology (PCOM). This disease is usually accompanied by insulin resistance (IR), obesity, and a series of metabolic disorders, which seriously affecting the reproductive health and overall health of patients (1). The prevalence of PCOS in women of childbearing age is as high as 6–12%, disrupting female reproductive hormone levels and resulting in anovulation and infertility (2, 3). Epidemiological studies have shown found that the incidence of PCOS in China was approximately 5.6%, and it was identified as the most common cause of ovulatory disorders in women of childbearing age (4). Clinically, affected PCOS patients commonly present with menstrual irregularities, endometrial hyperplasia, and abnormal uterine bleeding (5). Although the precise etiology of PCOS remains elusive, current evidence implicates genetic predisposition, psychological factors, and environmental influences as significant contributors (6). Due to limited understanding of its pathogenesis in PCOS, current therapeutic strategies primarily focus on symptomatic management and complication prevention (7). Furthermore, PCOS patients exhibit increased susceptibility to obstetric complications and experience higher rates of anxiety and depression, substantially impairing quality of life (8). Consequently, elucidating the underlying pathogenic mechanisms of PCOS and enabling early recognition are critical for developing targeted interventions.

Androgens exert pivotal effects on the female reproductive endocrine system. Animal studies confirmed their critical contribution to follicular development maintenance and fertility regulation (9). Mechanistically, androgens enhanced follicular recruitment and growth while up-regulating insulin-like growth factor 1 (IGF-1) expression, thereby modulating folliculogenesis (10). Moreover, substantial evidence indicated that excessive androgen levels constitute one of the core pathogenic mechanisms of PCOS, impairing follicular maturation and suppressing ovulation (11, 12). Exogenous androgen administration is a widely adopted method for establishing PCOS animal models, among which dehydroepiandrosterone (DHEA) is the reagent of first choice (13). As an important adrenal-derived androgen precursor, continuous subcutaneous administration of DHEA in prepubertal female mice can induce the formation of a large number of cystic follicles and elevated serum testosterone (T) levels, thereby leading to follicular developmental disorder, ovulatory dysfunction, and polycystic ovarian morphological changes (14). Hormone levels in PCOS patients are highly clinical heterogeneous, and HA is considered a distinguishing characteristic for the diagnosis of PCOS (15). In PCOS patients, HA inhibits follicular growth and maturation, leading to anovulatory infertility (16). HA is considered to be the core pathological component in PCOS pathogenesis and occurs in approximately 80% of PCOS patients (17). Multiple studies have indicated that one of the core pathologies of PCOS is IR and abnormalities in the insulin signaling pathway. These defects not only affect ovarian steroid production but also exacerbate metabolic disorders and promote a hyperandrogenic state (18–22). Furthermore, enhanced steroidogenesis by ovarian theca cells can lead to excessive T production, while alterations in sex hormone–binding globulin (SHBG) levels further influence the ratio of free androgens, which is closely associated with the clinical hyperandrogenism in PCOS (23–25). Nevertheless, the mechanistic role of hyperandrogenism in promoting metabolic comorbidities in PCOS remains incompletely defined. Current anti-androgen therapies yield suboptimal efficacy with significant adverse effects, underscoring the need to identify novel therapeutic targets for androgen regulation.

Recently, with the maturation of sequencing technologies and the widespread adoption of bioinformatics techniques, they have increasingly become one of the primary approaches for exploring the pathogenesis and etiology of diseases (26, 27). They have not only accelerated the process of disease diagnosis and treatment, but also provided robust support for the fields of individualized medicine, genetic disease screening and gene therapy. In addition, integrative biology technology enables in-depth analysis and comparison of diseases at the genetic level, facilitating more intuitive discovery of the genetic changes associated with the occurrence of diseases and revealing the pathogenesis of diseases, thus providing precise targets and strategies for the treatment and prevention of diseases (28, 29). Wang et al. (30) developed an IP scoring system using the GSVA method to predict the treatment response of solid tumor patients to immune checkpoint inhibitors (ICI). They found that patients in the high IP score group exhibited a higher ICI treatment response rate, providing a novel approach for exploring the prognosis of solid tumors. Zhu et al. (31) adopted bioinformatics approaches to investigate the prognostic value of N-glycan biosynthesis (NGB) in low-grade glioma (LGG), and identified that among the 22 NGB-related prognostic genes, *MGAT1* was the gene most strongly associated with poor prognosis of LGG, while *TUSC3* was the gene correlated with favorable prognosis of LGG. A study screened for co-expressed genes in PCOS and endometrial carcinoma (EC) by mining available sequencing data to further explore their roles in EC prognosis and treatment, revealing aberrant *IGF2* expression as a potential contributor to EC progression (32). Similarly, bioinformatic analyses have elucidated the regulatory role of m6A regulators in PCOS pathogenesis, suggesting novel gene-targeted therapeutic avenues in PCOS (33). Crucially, current bioinformatics studies have rarely investigated the correlation between androgen levels and the pathogenesis of PCOS. In this study, we identified androgen-related genes (ARGs) through integrated analysis of PCOS sequencing data, elucidating androgen-mediated mechanisms in PCOS development via bioinformatics analysis, aiming to propose novel directions for the clinical management of PCOS.

## 2 Materials and methods

### 2.1 Data collection and processing

We retrieved 26 healthy samples and 34 samples with PCOS from the GEO database (GSE34526, GSE80432, GSE95728, GSE124226, and GSE137684) (34–36). The inclusion criteria were as follows: (1) at least 5 samples per dataset; (2) Clinical information of PCOS patients and healthy controls was available in the datasets; (3) The publicly available raw data were derived from the bulk transcriptomic expression profiling. The exclusion criteria were: (1) Presence of other endocrine or metabolic disorders, including congenital adrenal hyperplasia, Cushing's syndrome, and androgen-secreting tumors; (2) Non-human samples or *in vitro* cell lines; (3) Mixed or undefined tissue origin. The detailed information of datasets is displayed in Supplementary Table 1. Based on previous studies, 91 ARGs were extracted from the Gene Set Enrichment Analysis (GSEA) website (http://www.gsea-msigdb.org/gsea/index.jsp), shown in Supplementary Table 2. First, the log2 transformation was performed to preprocess the raw data, and further quantile normalization was applied to minimize heterogeneity between microarray studies from different sequencing platforms. Batch effects were corrected by applying the “ComBat” algorithm implemented in the R package “sva.” Subsequently, normalization, batch correction, and differential expression analysis were applied for the five datasets via the “limma” and “sva” packages (*P* < 0.05), and visualized in boxplot (37).

### 2.2 Function enrichment analysis

To elucidate the biological functions of the differentially expressed ARGs, we performed Gene Ontology (GO) and Kyoto Encyclopedia of Genes and Genomes (KEGG) enrichment analyses using the R package “clusterProfiler” (*P* < 0.05) (38). The GO framework comprises three domains: molecular function (MF), biological process (BP), and cellular component (CC), providing a comprehensive gene annotation system for functional characterization.

### 2.3 Identification of hub ARGs

Protein-protein interaction (PPI) networks represent interconnected proteins that collectively participate in diverse biological processes (39). To identify hub genes, a PPI network was constructed based on differentially expressed ARGs. The STRING online database was utilized to construct the PPI network, which was screened with the lowest interaction score > 0.4. Subsequently, least absolute shrinkage and selection operator (LASSO) regression and random forest (RF) algorithms were implemented using the “glmnet” and “randomForestSRC” R packages to identify pivotal ARGs. Ultimately, hub ARGs associated with PCOS were determined by integrating the results from these three computational approaches.

### 2.4 Development of a nomogram for PCOS patients

A nomogram is a statistical tool that enables quantitative and graphical representation of prediction indicators based on logistic or Cox regression models (40). This method facilitates a more intuitive assessment of clinical outcomes. And we constructed a nomogram to forecast the incidence of PCOS using these hub ARGs. The receiver operating characteristic (ROC) curve and area under the curve (AUC) were calculated to determine the accuracy of hub ARGs for PCOS.

### 2.5 Classification of ARG molecular subtypes

Consensus clustering analysis was performed to produce unsupervised ARG molecular clusters with maximum *K* = 9 via the “ConsensusClusterPlus” package of R (41). To ensure the robustness of classification, we verified the clustering process 1,000 times in the cohort. Subsequently PCOS patients were classified into distinct molecular subtypes (*K* = 2–9) for further analysis. Next, the distributional differences of ARG subtypes were shown by principal component analysis (PCA). To identify differentially expressed genes (DEGs) from different ARG clusters, we conducted the “limma” package with *P* < 0.05 and |log2FC| > 1.

### 2.6 Assessment of immune features and gene expression in distinct subtypes

Single-sample gene set enrichment analysis (ssGSEA) was employed to quantify immune cell infiltration levels in PCOS samples (42). Additionally, we screened genes that are involved in the development of PCOS from the GeneCards (http://www.genecards.org/) for further analysis. To evaluate associations between different subtypes and the expression levels of these genes, we adopted differential expression analysis via R package “limma,” and the results were presented in boxplots.

### 2.7 Animal model construction

A total of twelve 3-week-old female SPF C57BL/6 mice were purchased from Sibeifu (Beijing) Biotechnology Co., Ltd. This study was approved by the Animal Welfare and Ethics Committee of Chengdu University of Traditional Chinese Medicine (2025002). After arrival, the animals were given adaptive feeding for 5 days, during which they were fed with basic feed (10 kcal% fat) and had free access to food and water.

After the adaptive feeding, the twelve mice were randomly divided into the control group (*n* = 6) and the PCOS group (*n* = 6). Mice in the PCOS group were subcutaneously injected with DHEA (Sigma, USA), 6 mg DHEA per 100 g body weight, once a day, for 21 consecutive days (43). Mice in control group received the equal volume of sesame oil as a control. Disruption of the estrous cycle in mice indicated the success of the PCOS model establishment (44). On the day after the completion of model establishment, all mice were sacrificed for the collection of serum and ovarian tissues. The bilateral ovaries of the mice were quickly removed, with one ovary fixed in 4% polyformaldehyde solution and the other side was rapidly placed in liquid nitrogen, then transferred to a −80 C refrigerator for storage until further analysis.

### 2.8 Assessment of estrous cycle

Starting on day 10 of modeling, vaginal smears were collected from mice in both groups daily at 9:00 a.m. until the end of modeling period. The estrous cycle phase of each mouse was determined by analyzing the cell types and morphology observed under the optical microscope to assess whether modeling was successful (43).

### 2.9 Serum measurement

The expression of T in serum was detected by enzyme-linked immunosorbent assay (ELISA) kit (Ruixin Biology, Quanzhou, China). All procedures were strictly performed according to the manufacturer's instructions.

### 2.10 Ovarian morphology

The detailed staining protocol used in this study was referenced from previous research (45). After the ovarian tissues were fixed in 4% paraformaldehyde (Biosharp, Beijing, China) for 24 h, they were sequentially subjected to gradient dehydration, paraffin embedding, paraffin sectioning (4 μm in thickness), and dewaxing. Finally, the ovarian sections were stained using the Hematoxylin-Eosin Staining Kit (Solarbio, Beijing, China), and the morphological changes of the ovaries were observed under microscope.

### 2.11 Quantitative real-time PCR (RT-qPCR) analysis

Following the experimental protocols, total RNA was extracted from mouse ovarian tissue samples. The cDNA synthesis was performed using the Transcriptor First Strand cDNASynthesis Kit (Roche, Germany) for reverse transcription. RT-qPCR was conducted with Stormstar SybrGreen Master Mix (DBI Bioscience, Germany). β-actin served as the internal reference gene. All primers were synthesized by SinoGene Biotechnology (Shanghai, China), with specific primer sequences listed in Supplementary Table 10.

### 2.12 Statistical analysis

R was used for bioinformatics analysis (version 4.1.3). The packages used were as follows: rms, rmda, GEOquery, limma, sva, clusterProfiler, org.Hs.eg.db, enrichplot, ggplot2, glmnet, randomForest, e1071, kernlab, caret, venn, ConsensusClusterPlus, pheatmap, reshape2, GSEABase, GSVA, VennDiagram, circlize, RColorBrewer, dplyr, ComplexHeatmap, and pROC. GraphPad Prism 8 was employed for data analysis and visualization. Student's *t*-test was applied for inter-group comparisons. *P* < 0.05 indicated statistically significant differences.

## 3 Results

### 3.1 Identify differentially expressed ARGs and perform enrichment analysis

**Figure 1:**
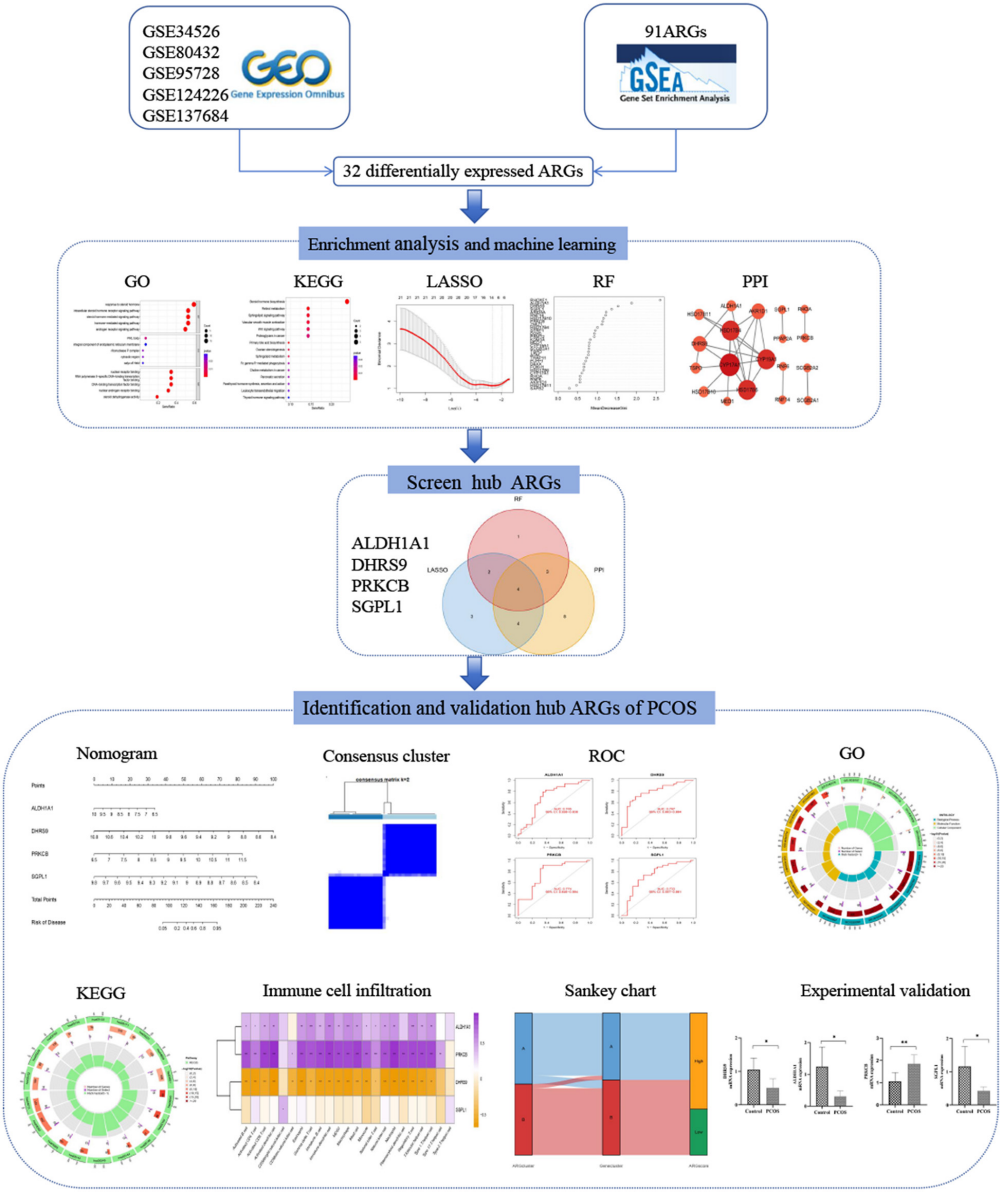
Study design flow chart.

**Figure 2:**
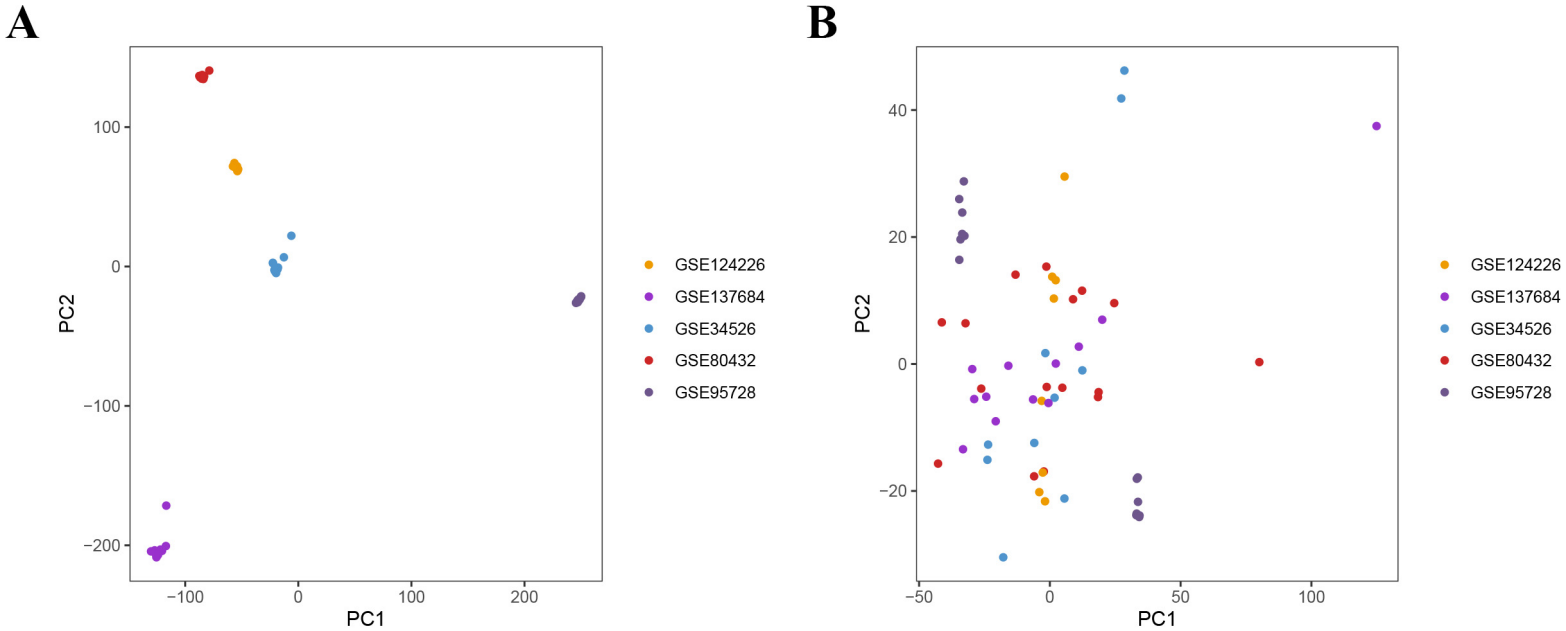
Merging and PCA processing of five datasets. **(A)** before the removal of the batch effect. **(B)** after the removal of the batch effect.

**Figure 3:**
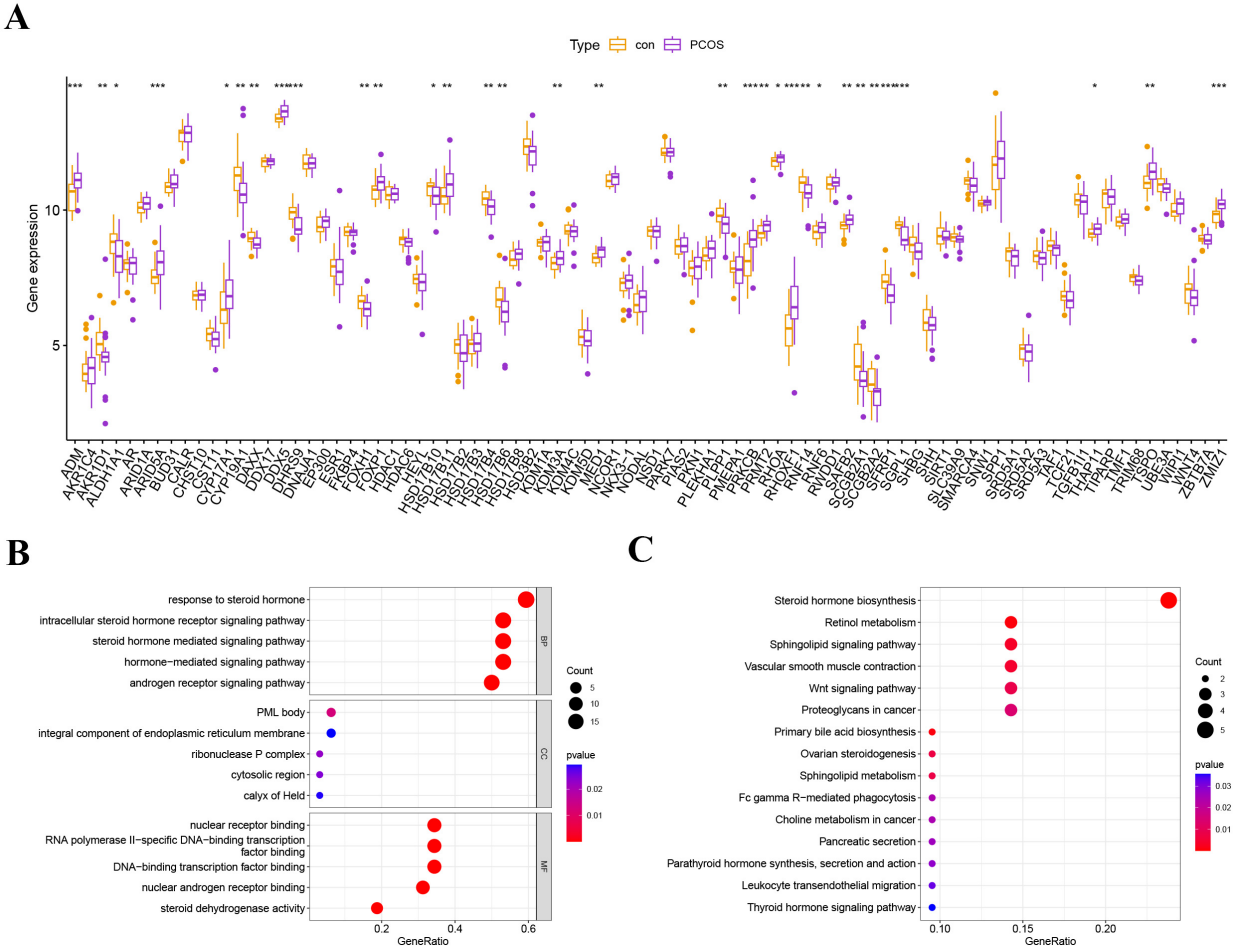
Expression analysis and functional enrichment of differentially ARGs. **(A)** Expression levels of 32 differentially expressed ARGs between healthy control and PCOS patients. **(B)** The GO analysis for 32 differentially expressed ARGs. **(C)** The KEGG analysis for 32 differentially expressed ARGs.

The workflow of the study is shown in Figure 1. To eliminate batch effects from the dataset, we employed the “sva” R package. PCA was used to assess the overall sample distribution and the effectiveness of normalization and batch effect correction prior to downstream analysis (Figures 2A, B). We found a total of 32 differentially expressed ARGs, with the expression shown in Figure 3A. Additionally, we investigated the biological mechanisms of differentially expressed ARGs for PCOS through GO and KEGG enrichment analysis. The GO results revealed that BP is linked to androgen receptor signaling pathway, intracellular steroid hormone receptor signaling pathway, and steroid hormone-mediated signaling pathway; CC is related to PML body, ribonuclease P complex, and cytosolic region; and MF is connected to nuclear androgen receptor binding, nuclear receptor binding, and RNA polymerase II-specific DNA-binding transcription factor binding (Figure 3B; Supplementary Table 3). KEGG pathway analysis revealed that differentially expressed ARGs were main enriched in steroid hormone biosynthesis, retinol metabolism, sphingolipid signaling pathway, Wnt signaling pathway, and ovarian steroidogenesis (Figure 3C; Supplementary Table 4).

### 3.2 Recognize hub ARGs

**Figure 4:**
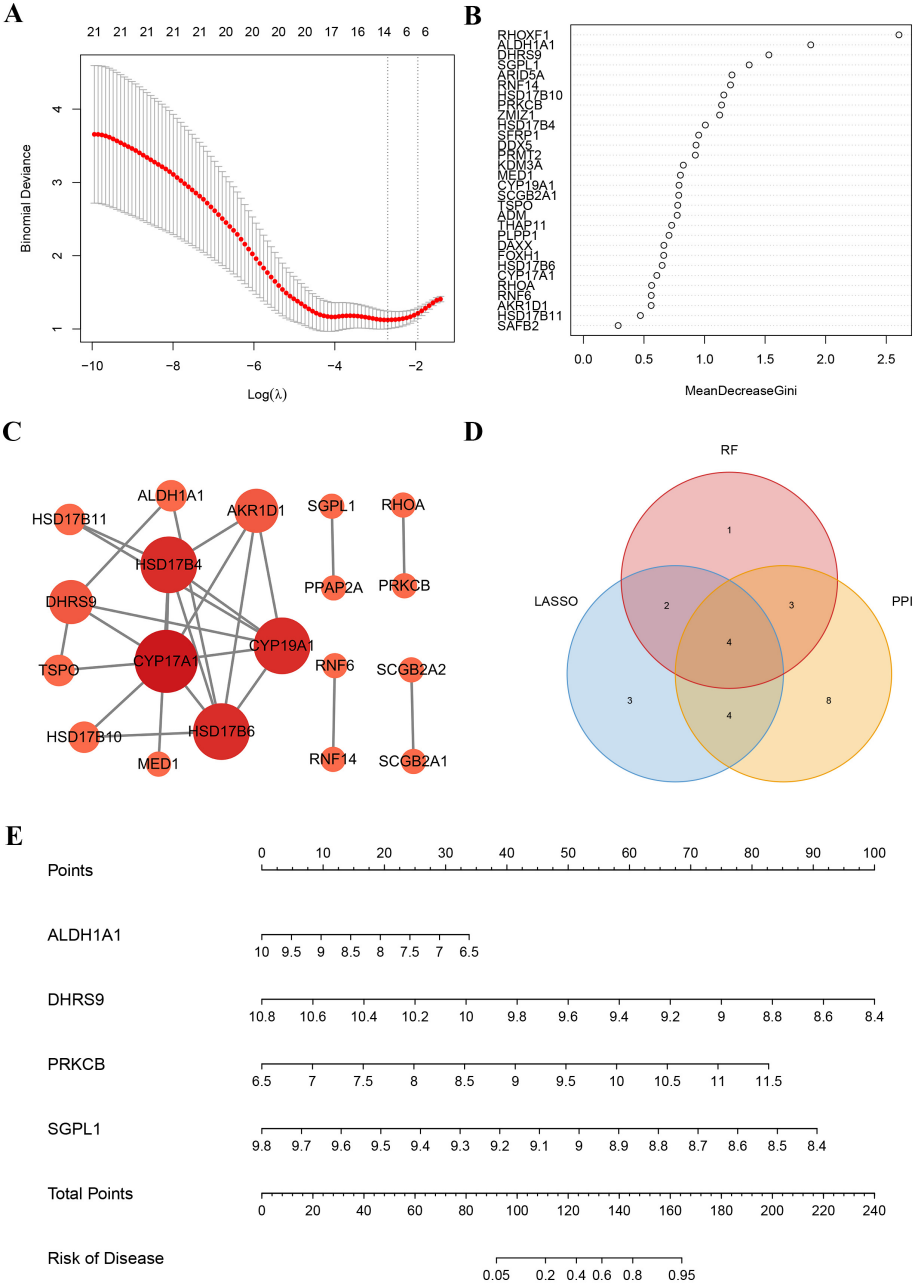
Machine learning selects the hub ARGs of PCOS. **(A)** LASSO coefficient profiles of 13 important ARGs. **(B)** RF algorithm result of 10 key ARGs. **(C)** The PPI network of the 19 critical ARGs. **(D)** Venn diagram showing hub ARGs (*ALDH1A1, DHRS9, PRKCB*, and *SGPL1*) shared by LASSO, RF, and PPI network. **(E)** A nomogram was utilized to predict the correlation between the expression levels of the hub ARGs and the occurrence of PCOS.

We identified 13 vital ARGs using the LASSO method (Figure 4A), 10 key ARGs by the RF method (Figure 4B), and 19 key ARGs via the PPI network (Figure 4C). Integration of these results yielded four hub ARGs (*ALDH1A1, DHRS9, PRKCB*, and *SGPL1*) associated with PCOS (Figure 4D).

### 3.3 Construct a nomogram for PCOS

Based on the four hub ARGs, we built a nomogram to predict the incidence of PCOS (Figure 4E). As illustrated in Supplementary Figure 1, the ROC curve analysis demonstrated strong discriminatory performance, with AUC values of 0.705 (*ALDH1A1*), 0.787 (*DHRS9*), 0.774 (*PRKCB*), and 0.733 (*SGPL1*), supporting their utility as predictive biomarkers.

### 3.4 Classify ARG molecular subtypes

**Figure 5:**
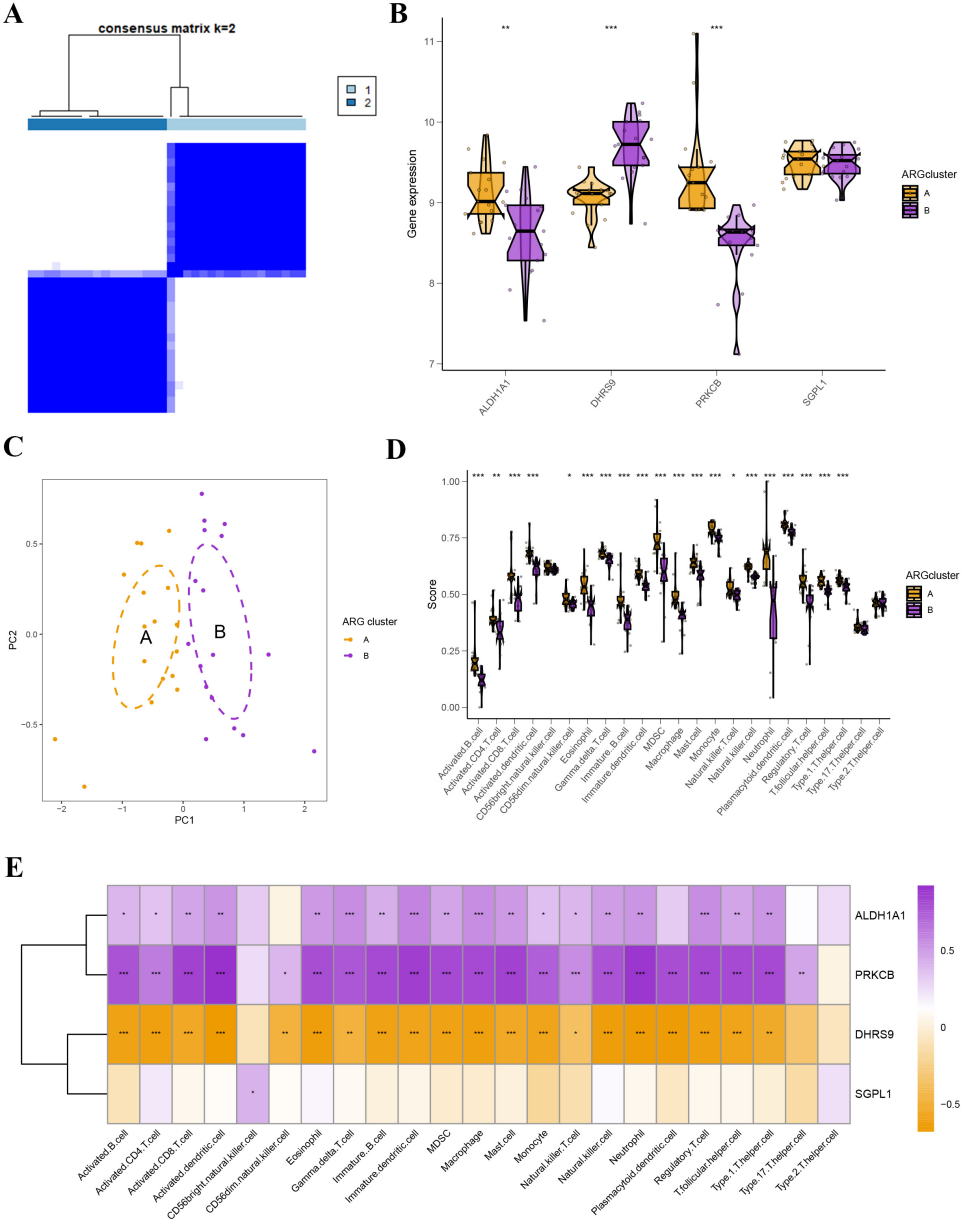
Identification and enrichment analysis of ARG subtypes. **(A)** Consensus matrices of the 4 hub ARGs (*k* = 2). **(B)** The expression levels of 4 hub ARGs between ARG clusters A and B. **(C)** The expression profiles of ARG clusters A and B. **(D)** Immune function analysis between ARG clusters A and B. **(E)** Correlation analysis between infiltrating immune cells and 4 hub ARGs.

The ARG clusters A and B were detected by consensus clustering method based on the four hub ARGs (Figure 5A; Supplementary Figure 2). In Figure 5B, the expression levels of *ALDH1A1, DHRS9*, and *PRKCB* were statistically differences in different ARG clusters. And PCA analysis proved the outstanding intergroup distribution of PCOS patients in ARG cluster A and B (Figure 5C). As shown in Figure 5D, higher levels of immune cell infiltration were observed in the ARG cluster A group compared to the ARG cluster B. Furthermore, we further explored the correlation between different hub ARGs and immune cell infiltration levels. Figure 5E found that four hub ARGs were significantly correlated with the 23 types of immune cells. *ALDH1A1* was significantly positively correlated with immature dendritic cell and macrophage, *PRKCB* was dramatically positively related to activated dendritic cell and neutrophil, and *SGPL1* was positively associated with CD 56 bright natural killer cell and Type 2 T helper cell. While *DHRS9* was negatively linked to plasmacytoid dendritic cell and eosinophil.

### 3.5 Identify DEGs of ARG clusters and analysis the immune microenvironment

**Figure 6:**
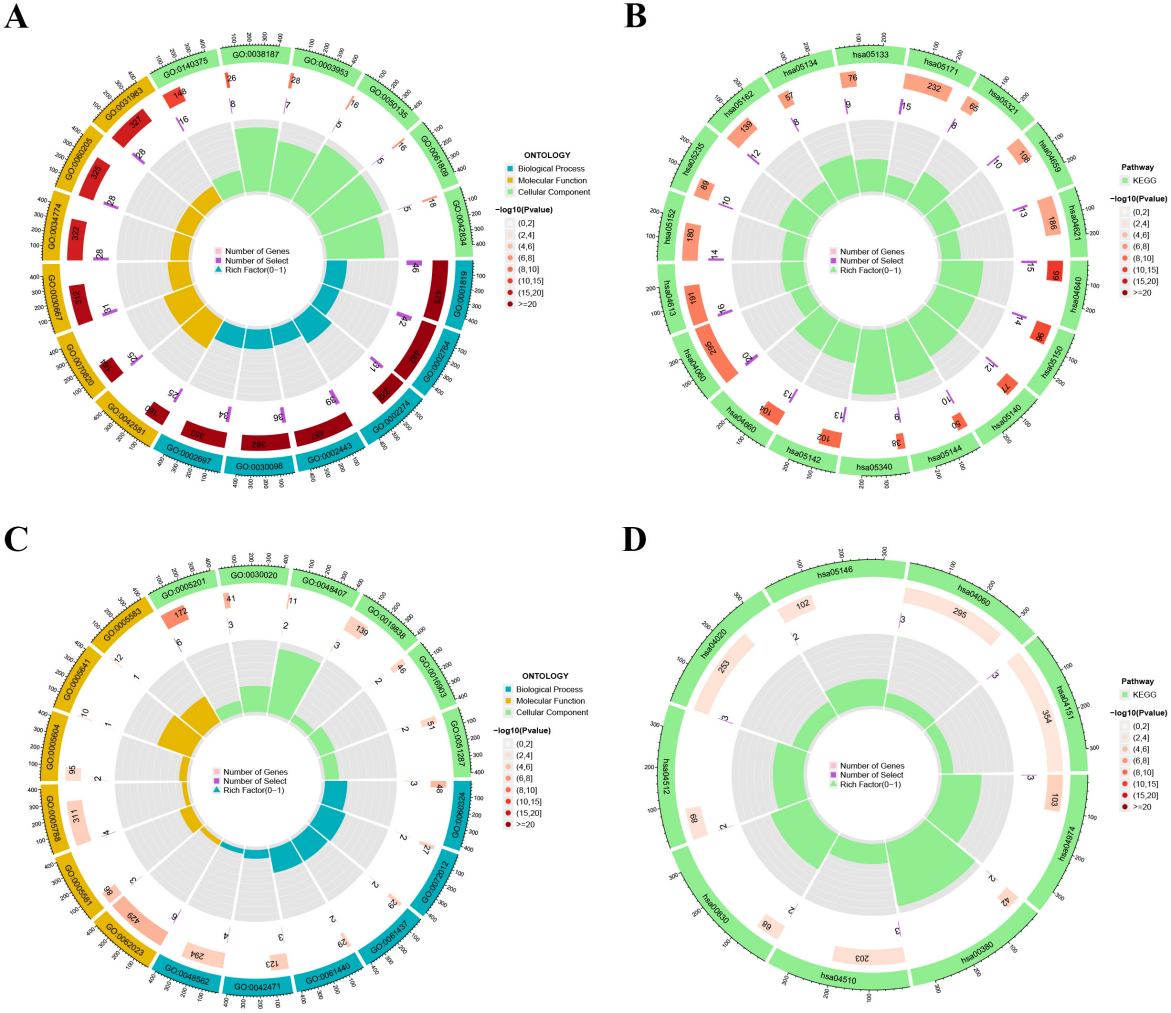
The enrichment analysis of different ARG clusters. **(A, B)** The GO and KEGG analysis for ARG cluster A. **(C, D)** The GO and KEGG analysis for ARG cluster B.

We screened DEGs of ARG clusters using “limma” package with *P* < 0.05 and |log2FC| > 1. In total, we screened 295 DEGs between ARG clusters A and B (Supplementary Table 5). Subsequently, we analyzed the functional enrichment of DEGs in different ARG clusters. The GO analysis results of ARG cluster A showed that BP was connected to positive regulation of cytokine production, immune response-regulating signaling pathway, and myeloid leukocyte activation; CC was related to specific granule, tertiary granule, and secretory granule membrane; and MF was linked to immune receptor activity, pattern recognition receptor activity, and NAD^+^ nucleosidase activity (Figure 6A; Supplementary Table 6). Additionally, the KEGG result of ARG cluster A suggested predominant enrichment in the hematopoietic cell lineage, staphylococcus aureus infection, primary immunodeficiency, T cell receptor signaling pathway and cytokine-cytokine receptor interaction (Figure 6B; Supplementary Table 7). The GO results of ARG cluster B indicated that BP was correlated to glomerulus vasculature development, and renal system vasculature development; CC was connected to collagen-containing extracellular matrix, collagen trimer, and endoplasmic reticulum lumen; and MF was associated with extracellular matrix structural constituent, cytokine activity, and platelet-derived growth factor binding (Figure 6C; Supplementary Table 8). KEGG analysis highlighted the ARG cluster B mainly enriched in protein digestion and absorption, retinol metabolism, ECM-receptor interaction, cytokine-cytokine receptor interaction, and PI3K-Akt signaling pathway (Figure 6D; Supplementary Table 9).

### 3.6 Classify gene molecular subtypes and assess the immune microenvironment

**Figure 7:**
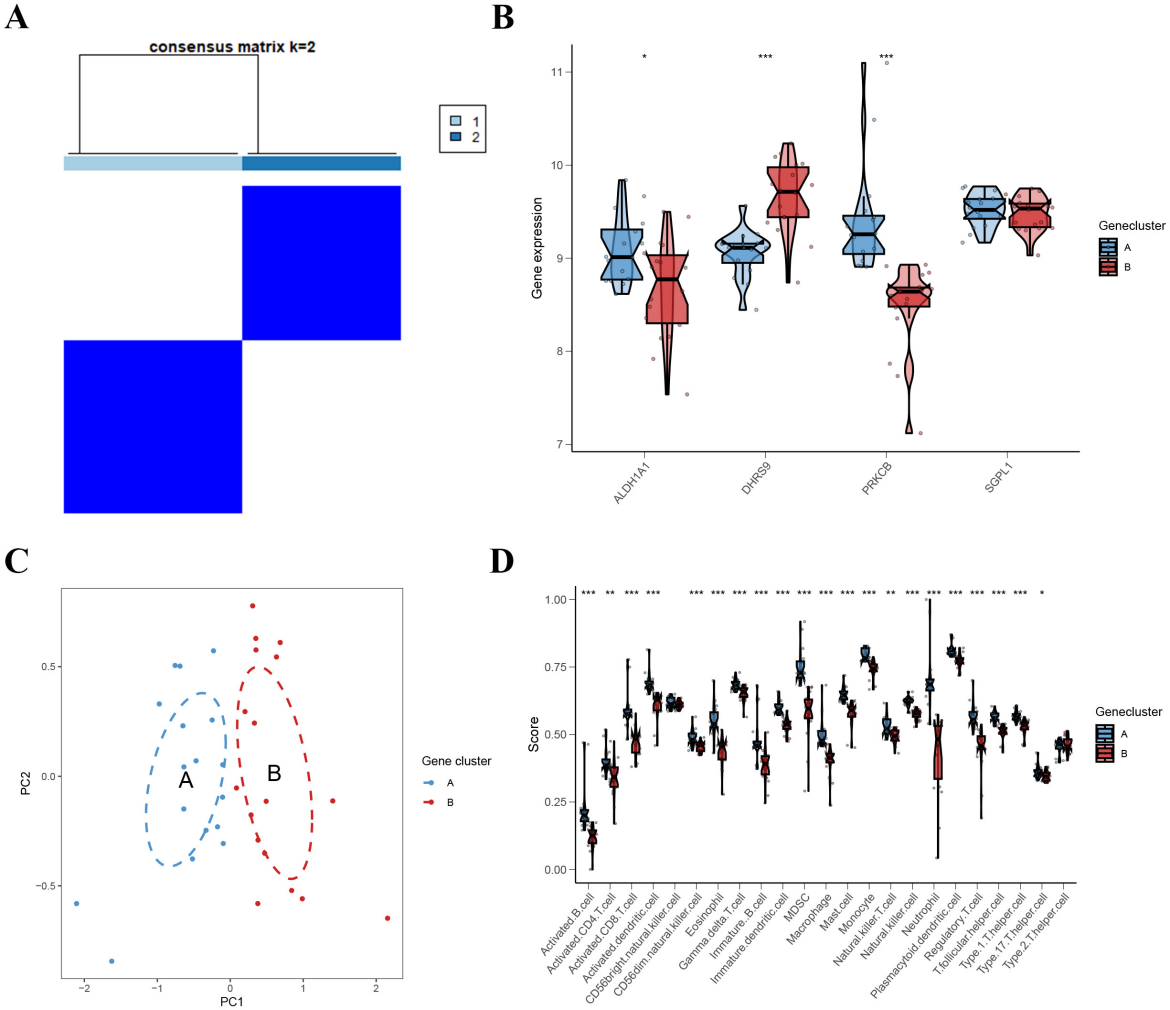
Identification and enrichment analysis of gene clusters. **(A)** Consensus matrices of 359 DEGs (*k* = 2). **(B)** Differential expression of the 4 ARGs in gene clusters A and B. **(C)** The expression profiles of gene clusters A and B. **(D)** Immune function analysis between gene clusters A and B.

Unsupervised consensus clustering analysis was performed on DEGs to acquire distinct gene clusters and we discovered two gene clusters (gene clusters A and B) (Figure 7A; Supplementary Figure 3). Expression of *ALDH1A1, DHRS9*, and *PRKCB* differed significantly between gene clusters (Figure 7B). As shown in Figure 7C, PCA results confirmed significant distribution patterns of PCOS patients in gene cluster A and B.And the abundance of immune cell infiltration in the gene cluster A was markedly higher than gene cluster B (Figure 7D).

### 3.7 Correlation analysis between immune features and gene expression in different clusters

**Figure 8:**
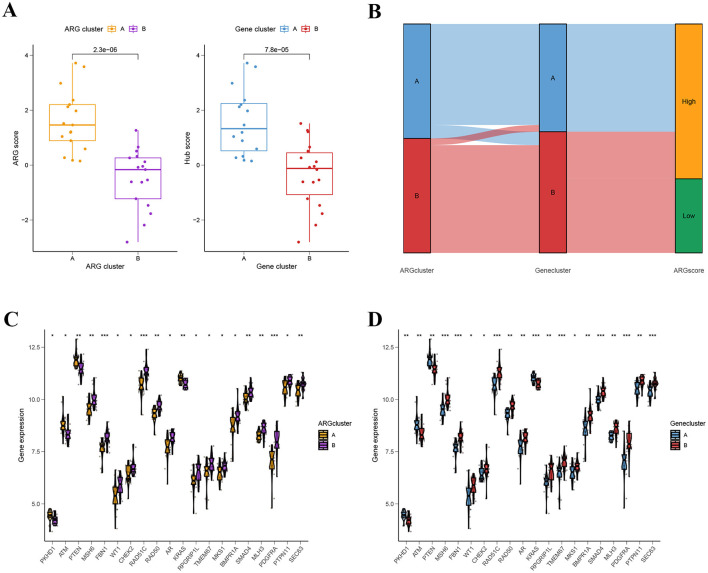
Identification and comparison of different gene subtypes. **(A)** Differences in ARG score between ARG clusters and differences in ARG score between gene clusters. **(B)** Sankey Chart of subtype distributions between ARG cluster, gene cluster, and ARG score. **(C, D)** *PKHD1, ATM, PTEN*, and *KRAS* were over-expressed, while *MSH6, FBN1, WT1, CHEK2, RAD51C, RAD50, AR, RPGRIP1L, TMEM67, MKS1, BMPR1A, SMAD4, MLH3, PDGFRA, PTPN11*, and *SEC63* were down-regulated in ARG cluster A and gene cluster A.

We found significant differences in ARGs scores between the two ARG clusters and the two gene clusters (Figure 8A). Interestingly, ARG cluster A or gene cluster A had considerably higher ARG scores compared to ARG cluster B or gene cluster B, suggesting ARG cluster A or gene cluster A might be more closely associated with pathological changes in PCOS. Figure 8B showed the distribution of PCOS patients in the two ARG clusters, two gene clusters, and two ARG score groups. Moreover, we assessed the expression levels of genes associated with the pathogenesis of PCOS in different clusters. As shown in Figures 8C, D, *PKHD1, ATM, PTEN*, and *KRAS* were over-expressed, while *MSH6, FBN1, WT1, CHEK2, RAD51C, RAD50, AR, RPGRIP1L, TMEM67, MKS1, BMPR1A, SMAD4, MLH3, PDGFRA, PTPN11*, and *SEC63* were down-regulated in ARG cluster A and gene cluster A.

### 3.8 Validation of hub ARGs in PCOS animal models

**Figure 9:**
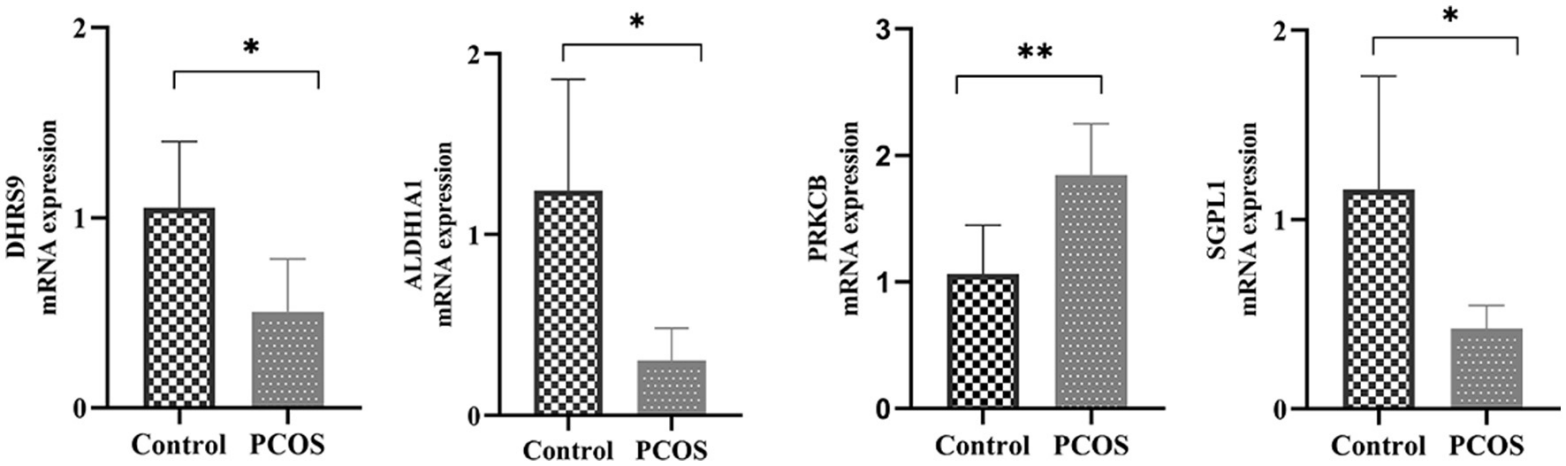
Validation of the mRNA expression of 4 hub ARGs in PCOS mice. *n* = 6.*P < 0.05; **P < 0.01.

To further validate the expression levels of these hub ARGs in the progression of PCOS, we established the PCOS model in mice. Compared with control group, PCOS group exhibited disrupted estrous cycle patterns, with prolonged retention in the estrus phase. Ovarian sections from PCOS group revealed multiple large cystic follicles, reduced granulosa cell layers, and increased numbers of atretic follicles. Additionally, serum testosterone levels were significantly elevated in PCOS group (Supplementary Figure 4). RT-qPCR results showed that *DHRS9, SGPL1*, and *ALDH1A1* were significantly downregulated in PCOS group, while *PRKCB* was significantly upregulated (*P* < 0.05) (Figure 9). These findings were consistent with bioinformatics analysis results, demonstrating the reliability of the analysis.

## 4 Discussion

PCOS is a widespread reproductive endocrine disorder among women and represents the most common cause of anovulatory infertility in those of reproductive age (46). Abnormally raised androgen levels are a significant feature of endocrine disruption in PCOS, causing clinical manifestations such as hirsutism and acne (16). Excessive androgens make the ovaries function aberrantly in PCOS patients, inhibiting follicle growth and ultimately contributing to ovulation disorders (47). Presently, anti-androgen therapy is the generally treatment for patients with PCOS, and the clinical outcome is unsatisfactory due to the complex mechanisms of androgen production (48). Therefore, this study sought to investigate the role of androgens in PCOS by identifying candidate ARGs from PCOS patients through the publicly available GEO datasets, aiming to provide new targets for anti-androgen therapeutic strategies in PCOS.

As an essential component of the ovarian microenvironment, ovarian GCs play an indispensable role in follicular development and ovulation, and provide the material basis and spatial support for oocyte growth and maturation (49). Ovarian GCs are involved in the pathogenesis of PCOS by causing GCs dysfunction during early follicular growth through self-proliferation, apoptosis and hormone synthesis (50). Additionally, GCs occupied major positions in steroidogenesis and ovarian folliculogenesis, and their dysfunction has been implicated in the pathogenesis of reproductive disorders; thus, most of the PCOS studies have been carried out using ovarian GCs as experimental samples (51). Whole-gene transcriptome sequencing results revealed that GCs from control and PCOS patients had different IncRNA and mRNA profiles (52). Ovarian GCs from PCOS patients were found to possess multiple transcriptional and epigenetic changes indicating possible association with steroid hormone synthesis and metabolism pathways (53). Several studies have suggested that abnormal follicular development in PCOS patients might be connected with the dysfunction of ovarian GCs (54, 55). Collectively, ovarian GCs represent a reasonable entry point for investigating the underlying etiopathogenesis of PCOS.

In this study, we integrated multiple bioinformatics approaches, including LASSO regression, RF, and PPI network, to identify four hub ARGs (*ALDH1A1, DHRS9, PRKCB*, and *SGPL1*) involved in PCOS pathogenesis. We also constructed a nomogram for predicting the risk of PCOS based on these hub ARGs, which showed promising diagnostic performance with AUC values ranging from 0.70 to 0.78. Although external validation was not conducted due to sample size limitations, we performed internal cross-validation to evaluate the model's stability. We further validated their expression in the PCOS animal model, demonstrating consistency with our computational predictions. Our findings underscore the potential of these hub ARGs as biomarkers for PCOS diagnosis and provide insights into the molecular mechanisms underlying the disease. However, further clinical studies with larger sample sizes are needed to validate these biomarkers and explore their therapeutic potential.

*ALDH1A1* is a cytoplasmic enzyme belonging to the aldehyde dehydrogenase family. It exhibits diverse biological functions and has been found to exert crucial roles in physiological and pathological changes of various diseases (56, 57). Earlier studies showed that *ALDH1A1* has specific effects on androgens and performs a major role in endocrine metabolism (57). *ALDH1A1* expression was changed in endometrial samples from PCOS patients compared to healthy controls, suggesting that *ALDH1A1* could be a prospective risk factor for facilitating dysfunction of the endometrium in PCOS (58). Our result reconfirms the relevance of *ALDH1A1* to the pathogenesis of PCOS. Besides, *ALDH1A1* is recognized as a key biomarker of breast cancer stem cells and promotes breast cancer malignancy progression (59). The high level of *ALDH1A1* was positively correlated with poor prognosis in thyroid cancer, suggesting that it may be a potential prognostic gene for thyroid cancer (60). In ovarian cancer, *ALDH1A1* inhibitor reduced the number of its stem cells and moderately decreased the recurrence rate of ovarian cancer (61). *DHRS9* is a member of the short-chain dehydrogenase/reductase family. *DHRS9* was shown closely associated with abnormalities in ovarian development and function in PCOS (62). A study demonstrated that *DHRS9* was involved in the synthesis and metabolism of androgen in prostate cancer (63). Additionally, *DHRS9* may serve as a possible biomarker for human regulatory macrophages, offering potential strategies for cell-based immunotherapy (64). Furthermore, *DHRS9* was aberrantly expressed in various cancers, including pancreatic and colorectal cancer (65, 66). While the correlation between *DHRS9* and PCOS remains underexplored, our results provide new ideas for exploring the pathogenic mechanisms of PCOS.

*PRKCB* is a family member of protein kinase C, located on human chromosome 16, with transcriptional functions. Transcriptome sequencing of PCOS mouse oocytes revealed the involvement of *PRKCB* in metabolic pathways related to androgenic effects (67). Another sequencing result also found that *PRKCB* gene in the androgen signaling pathway was significantly down-regulated in granulosa cells, showing that *PRKCB* is closely linked to androgens (68). Earlier studies discovered that the epigenetic functions of *PRKCB* were depended on androgen signaling (69). In addition, several studies demonstrated significant associations between *PRKCB* and poor prognosis in various cancer, such as lung adenocarcinoma and pancreatic cancer (70, 71). *SGPL1*, encoded by 568 amino acids, is an irreversible degrading enzyme of sphingosine-1-phosphate (S1P) (72). Deficiency and mutations in *SGPL1* were found to cause the development of multiple metabolic defective disorders, such as congenital nephrotic syndrome and Charcot-Marie-Tooth neuropathy (73, 74). Additionally, *SGPL1* exerted oncogenic effects through activation of cancer-associated transcription factors. Located in regions of cancer-prone to mutations, *SGPL1* gene was involved in the regulation of tumor cell pathological processes (75, 76). Interestingly, another study confirmed that *SGPL1* promoted tumorigenesis by the glucose metabolism pathway (77). Recent studies proved that *SGPL1* could affect the development of germ cells. Knockdown of *SGPL1* in mice inhibited granulosa cell proliferation, causing the failure of follicle development and ultimately stunting oocyte development (78). Although the present work is based on integrative transcriptomic analyses, our KEGG pathway results provide a biologically grounded context for mechanistic inference. In the study, the KEGG enrich pathways observed in our ARGs, including steroid hormone biosynthesis, ovarian steroidogenesis, retinol metabolism, and sphingolipid signaling, are highly relevant to ovarian steroidogenic programming and androgen homeostasis. Based on the above findings, the four hub ARGs identified (*ALDH1A1, DHRS9, PRKCB*, and *SGPL1*) may influence androgen metabolism through partially routes involving ovarian microenvironmental remodeling and steroidogenesis.

Numerous studies have reported the close association of PCOS with the chronic low-grade inflammatory status (79). On the one hand, the inflammatory markers were abnormally highly expressed in the serum of PCOS patients, indicating that there is a significant inflammatory response in the patient's body (80). On the other hand, the long-term inflammatory state of PCOS may be an important risk factor for early pregnancy loss in pregnant women (81). In the present study, GO, KEGG, and GSEA enrichment analysis observed that the analysis results were mainly enriched in genes and signaling pathways associated with immune inflammation. Besides, we found that levels of immune cell infiltration were also relatively high in PCOS patients, suggesting that PCOS patients might be more susceptible to inflammatory responses, concomitant tissue damage, and other pathological changes (82). Meanwhile, the four hub ARGs were closely correlated with immune pathways and inflammatory responses. *ALDH1A1* deficiency markedly changed the expression of pro-inflammatory cytokines in the blood of obese mice (83). *DHRS9* serves as a biomarker for macrophages with immunosuppressive activity (64). *PRKCB* was proven to be involved in the pathomechanism of systemic lupus erythematosus (84). Basic studies observed that the lack of *SGPL1* in gut epithelium accelerated tumor growth, enhanced *STAT3* activity, and increased inflammatory cytokines levels (85). Overall, there is a robust relationship between PCOS and the chronic low-grade inflammatory state.

However, our study still had a few limitations. First since the relatively small number of sequencing samples in the individual datasets, we integrated five datasets for analysis, which may have biased the analysis results. To avoid possible analytical bias, we performed batch effect on all datasets to normalize and unify the expressions of the datasets. Second, due to the difficulty in collecting clinical samples from both PCOS patients and healthy individuals, we only used animal experiments to preliminarily validate the bioinformatics results. Future studies will endeavor to conduct clinical trials and *in vitro* experiments to further elucidate and validate the functions and roles of these target genes. inally, the lack of an external validation cohort limits the clinical applicability of our current model. Future validation using larger, independent cohorts combined with clinical parameters is therefore warranted.

## 5 Conclusion

In conclusion, we identified four hub ARGs (*ALDH1A1, DHRS9, PRKCB*, and *SGPL1*) in PCOS patients from GEO database using bioinformatics analysis. Additionally, we also constructed a PCOS animal model to validate the diagnostic performance of these hub ARGs. A reliable diagnostic and subtype classification model was built and verified for PCOS. Furthermore, functional enrichment analysis and immune cell infiltrate revealed the underlying role of androgen and inflammation in the pathogenesis of PCOS. Taken together, our findings deepen the current understanding of the mechanism underlying PCOS pathogenesis and provide new insights for PCOS diagnosis and clinical treatment.

## Data Availability

The original contributions presented in the study are included in the article/supplementary material, further inquiries can be directed to the corresponding authors.
