# Supplementary material for: Exploring the biological functions of PCOS: identifying hub androgen-related genes through bioinformatics
Source: Front Med (Lausanne). 2026 Mar 19;13:1693216. doi: 10.3389/fmed.2026.1693216 (PMC13043399; doi:10.3389/fmed.2026.1693216)
Supplement: Supplementary file 1 [file Table_1.docx]

Supplementary Table 1. The information of the GEO datasets.

| Datasets | Total sample | Control | PCOS | Organizational sources | Platform |
| --- | --- | --- | --- | --- | --- |
| GSE34526 | 10 | 3 | 7 | Granulosa cells | [GPL570](https://www.ncbi.nlm.nih.gov/geo/query/acc.cgi?acc=GPL570) |
| GSE80432 | 16 | 8 | 8 | Granulosa Cells | GPL6244 |
| GSE95728 | 14 | 7 | 7 | Granulosa cells | [GPL16956](https://www.ncbi.nlm.nih.gov/geo/query/acc.cgi?acc=GPL16956) |
| GSE137684 | 12 | 4 | 8 | Granulosa cells | [GPL17077](https://www.ncbi.nlm.nih.gov/geo/query/acc.cgi?acc=GPL17077) |
| GSE124226 | 8 | 4 | 4 | Abdominal adipose stem cells | [GPL570](https://www.ncbi.nlm.nih.gov/geo/query/acc.cgi?acc=GPL570) |
