# Supplementary material for: Exploring the biological functions of PCOS: identifying hub androgen-related genes through bioinformatics
Source: Front Med (Lausanne). 2026 Mar 19;13:1693216. doi: 10.3389/fmed.2026.1693216 (PMC13043399; doi:10.3389/fmed.2026.1693216)
Supplement: Supplementary file 3 [file Table_3.docx]

**Table S10. Primers used for RT-qPCR**

| Primer | Sequences (5' → 3') | Product size（Bp） |
| --- | --- | --- |
| ALDH1A1 F： | ATACTTGTCGGATTTAGGAGGCT | 192 |
| ALDH1A1 R： | GGGCCTATCTTCCAAATGAACA |  |
| DHRS9 F： | ATGCTGTTTTGGTTGTTGGCT | 143 |
| DHRS9 R： | GTTCTGGCTGCTAAGTTTCCA |  |
| PRKCB F： | GTGTCAAGTCTGCTGCTTTGT | 141 |
| PRKCB R： | GTAGGACTGGAGTACGTGTGG |  |
| SGPL1 F： | CTGAAGGACTTCGAGCCTTATTT | 113 |
| SGPL1 R： | ACTCCACGCAATGAGCTGC |  |
| β-actin F： | TGAGCTGCGTTTTACACCCT | 231 |
| β-actin R： | TTTGGGGGATGTTTGCTCCA |  |
