# Supplementary figures and images for: Exploring the biological functions of PCOS: identifying hub androgen-related genes through bioinformatics
Source: Front Med (Lausanne). 2026 Mar 19;13:1693216. doi: 10.3389/fmed.2026.1693216 (PMC13043399; doi:10.3389/fmed.2026.1693216)

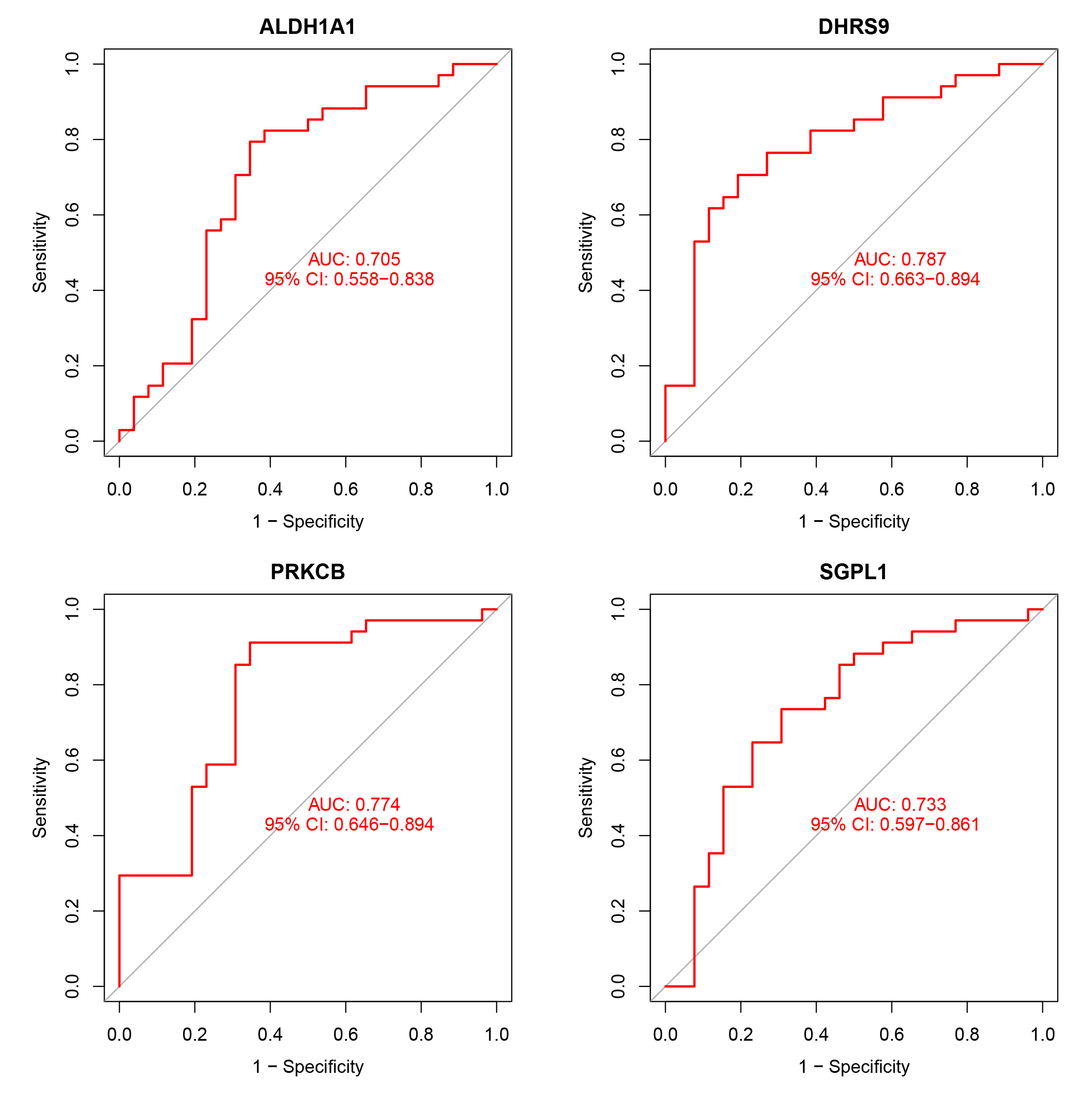

Supplement: Supplementary file 4 [file Image_1.tif]

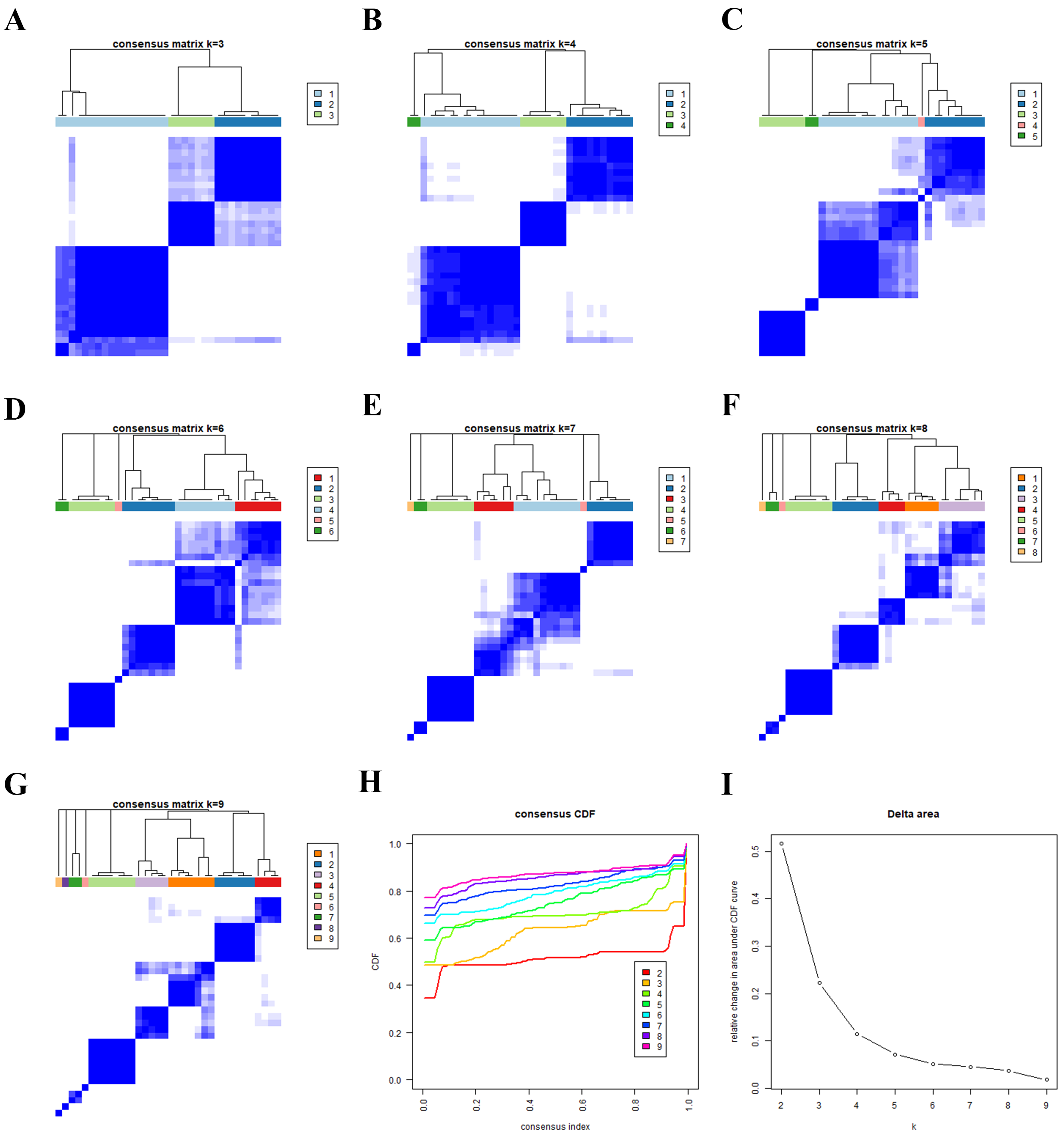

Supplement: Supplementary file 5 [file Image_2.tif]

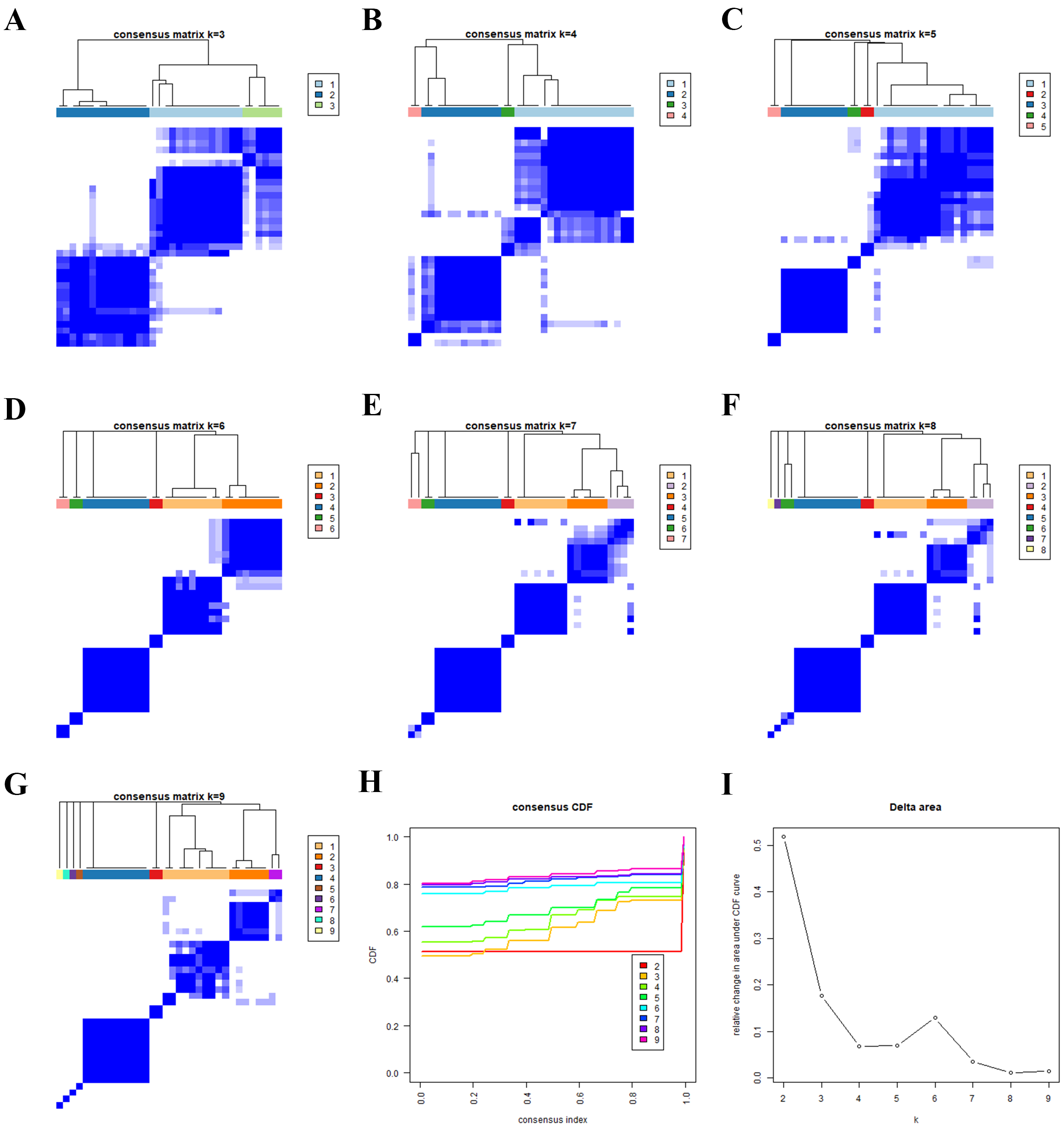

Supplement: Supplementary file 6 [file Image_3.tif]

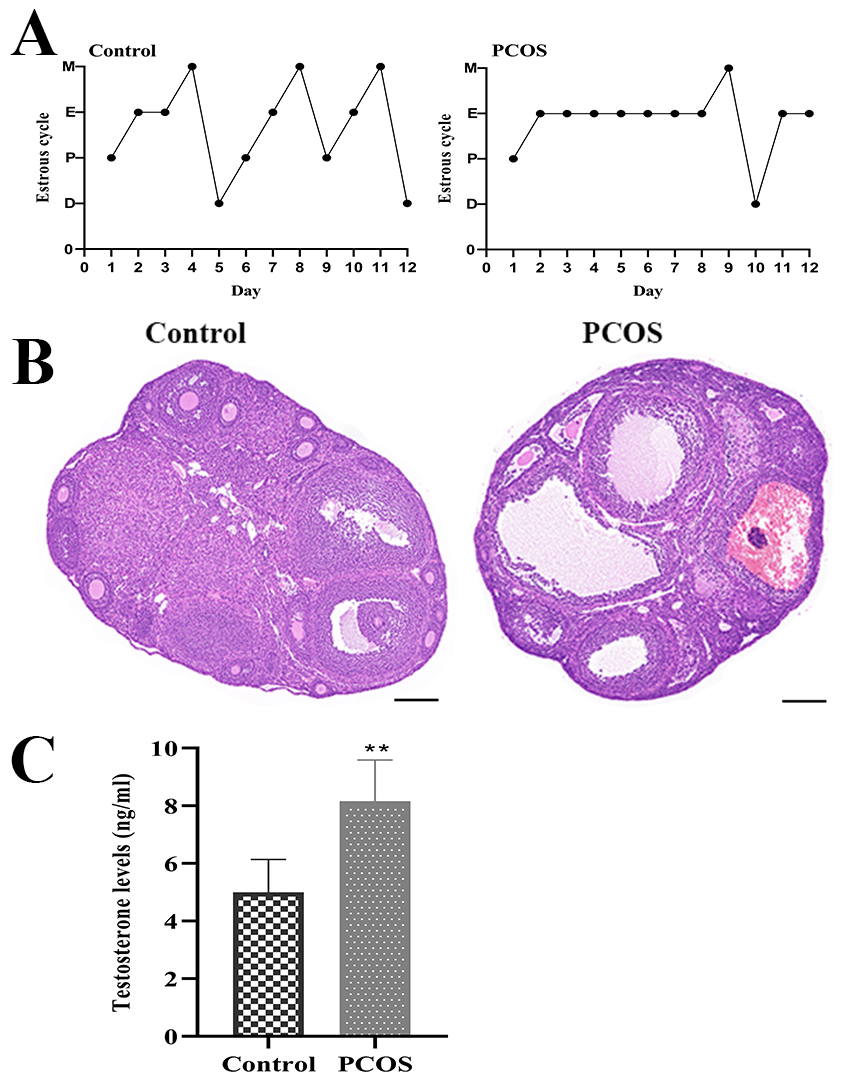

Supplement: Supplementary file 7 [file Image_4.tif]
